# A policy model of cardiovascular disease in moderate-to-advanced chronic kidney disease

**DOI:** 10.1136/heartjnl-2016-310970

**Published:** 2017-08-05

**Authors:** Iryna Schlackow, Seamus Kent, William Herrington, Jonathan Emberson, Richard Haynes, Christina Reith, Christoph Wanner, Bengt Fellström, Alastair Gray, Martin J Landray, Colin Baigent, Borislava Mihaylova

**Affiliations:** 1 Health Economics Research Centre, Nuffield Department of Population Health, University of Oxford, Oxford, UK; 2 Clinical Trial Service Unit and Epidemiological Studies Unit, Nuffield Department of Population Health, University of Oxford, Oxford, UK; 3 Division of Nephrology, Department of Medicine, University Hospital of Würzburg, Würzburg, Germany; 4 University Hospital, Uppsala, Sweden; 5 Medical Research Council Population Health Research Unit, Nuffield Department of Population Health, University of Oxford, Oxford, UK

**Keywords:** chronic kidney disease, cardiovascular risk, CKD progression, markov model, life expectancy

## Abstract

**Objective:**

To present a long-term policy model of cardiovascular disease (CVD) in moderate-to-advanced chronic kidney disease (CKD).

**Methods:**

A Markov model with transitions between CKD stages (3B, 4, 5, on dialysis, with kidney transplant) and cardiovascular events (major atherosclerotic events, haemorrhagic stroke, vascular death) was developed with individualised CKD and CVD risks estimated using the 5 years’ follow-up data of the 9270 patients with moderate-to-severe CKD in the Study of Heart and Renal Protection (SHARP) and multivariate parametric survival analysis. The model was assessed in three further CKD cohorts and compared with currently used risk scores.

**Results:**

Higher age, previous cardiovascular events and advanced CKD were the main contributors to increased individual disease risks. CKD and CVD risks predicted by the state-transition model corresponded well to risks observed in SHARP and external cohorts. The model’s predictions of vascular risk and progression to end-stage renal disease were better than, or comparable to, those produced by other risk scores. As an illustration, at age 60–69 years, projected survival for SHARP participants in CKD stage 3B was 13.5 years (10.6 quality-adjusted life years (QALYs)) in men and 14.8 years (10.7 QALYs) in women. Corresponding projections for participants on dialysis were 7.5 (5.6 QALYs) and 7.8 years (5.4 QALYs). A non-fatal major atherosclerotic event reduced life expectancy by about 2 years in stage 3B and by 1 year in dialysis.

**Conclusions:**

The SHARP CKD-CVD model is a novel resource for evaluating health outcomes and cost-effectiveness of interventions in CKD.

**Trial registration number:**

NCT00125593 and ISRCTN54137607; Post-results.

## Background

Chronic kidney disease (CKD) contributes significantly to the global burden of disease. The prevalence of CKD stages 3 and above in the adult UK population in 2009/2010 was 6% and is expected to rise with ageing populations and increasing prevalence of diabetes. Cardiovascular and all-cause mortality are increased in CKD,^1 2^ with most patients with CKD dying before reaching end-stage renal disease.^3^ Conversely, cardiovascular disease (CVD) is associated with increased risk of CKD progression.^4^ Policy models, used to evaluate long-term effectiveness and cost-effectiveness of interventions in CKD, need to account for this interdependence.

In published long-term CKD models,^5–7^ the cardiovascular risks have been based on Framingham risk equations, adjusted for an increased hazard with CKD^5 6^ or data from population with only mildly impaired renal function^7^; and the risk of CKD progression is either assumed independent from^5 7^, or is only partially adjusted for^6^, known risk factors. In two published models, dialysis and transplantation are not considered separately^5 6^ despite large differences in outcomes,^8^ and assessment of model performance is limited to comparisons with published data.^6 7^

We present a policy model in moderate-to-advanced CKD developed using the detailed individual participant data of the Study of Heart and Renal Protection (SHARP), which overcomes many of these limitations. The model allows for the interdependence between CKD and cardiovascular complications and simulates the progression of CKD, experience of cardiovascular events and death, health-related quality of life and healthcare costs. The model was validated internally, in several external datasets and against existing risk scores.

## Methods

### The Study of Heart and Renal Protection

Details of SHARP were reported previously.^9 10^ Briefly, patients with CKD aged ≥40 years with ≥2 previous measurements of serum or plasma creatinine ≥1.7 mg/dL (150 µmol/L) in men or ≥1.5 mg/dL (130 µmol/L) in women, but without life-limiting conditions, history of myocardial infarction or coronary revascularisation or functioning or planned kidney transplant, were eligible. At study entry, information was collected on sociodemographic characteristics (age, gender, ethnicity, number of children and adult dependants, highest level of education), physical measurements (body mass index (BMI), systolic and diastolic blood pressure), lipids (including total and high density lipoprotein (HDL) cholesterol)), other laboratory measurements (albumin, haemoglobin and phosphate), smoking status, urinary albumin:creatinine ratio (uACR), cause of kidney disease and history of CVD and diabetes. At study visits, scheduled at 2, 6, 12 months and every 6 months thereafter, information was recorded about myocardial infarctions, strokes, vascular procedures, hospital admissions and other serious adverse events.

### SHARP CKD-CVD lifetime outcomes model

The SHARP CKD-CVD decision analytic model includes a CKD submodel, simulating progression of CKD, and CVD submodel, simulating experience of fatal and non-fatal cardiovascular events and non-vascular death. The submodels are summarised below; further detail is provided in online supplementary item S1. For each participant, the annual risks of CVD and CKD endpoints were estimated using multivariate risk equations with a range of baseline characteristics and time-updated age, time since CKD diagnosis, CVD history (including within-trial events) and CKD status at end of previous year.

10.1136/heartjnl-2016-310970.supp1 Supplementary material 1

### CVD submodel

The annual risks of three nested composite cardiovascular endpoints were evaluated: (1) vascular death (ie, coronary, stroke or other vascular death), (2) vascular death or non-fatal major atherosclerotic event (ie, vascular death, myocardial infarction, non-haemorrhagic stroke or arterial revascularisation), and (3) vascular death or non-fatal major vascular event (ie, vascular death, myocardial infarction, any stroke or arterial revascularisation). Parametric proportional hazard multivariate survival models (Exponential, Weibull or Gompertz selected using the Akaike information criterion (AIC^11^) were estimated to support extrapolation over a patient’s lifetime.

### CKD submodel

For each year of follow-up, data collected during that year were used to categorise participants into one of five CKD states: CKD stage 3B (estimated glomerular filtration rate (eGFR): 30≤ eGFR <45 mL/min/1.73 m^2^), CKD stage 4 (15≤ eGFR <30 mL/min/1.73 m^2^), CKD stage 5 (eGFR <15 mL/min/1.73 m^2^) but not yet receiving renal replacement therapy (RRT: maintenance dialysis or kidney transplant), on maintenance dialysis, and with a kidney transplant.

In each year, participants not on RRT could progress to (or remain in) any CKD category, with transition probabilities estimated using multivariate multinomial logistic regression. For participants on dialysis, the probability of receiving a kidney transplant was estimated using logistic regression. Due to the small number of kidney transplants that failed between years of follow-up in SHARP, a constant annual probability of a transplant failing was estimated based on the overall failure rate observed in the study.

### SHARP CKD-CVD model structure

**Figure 1:**
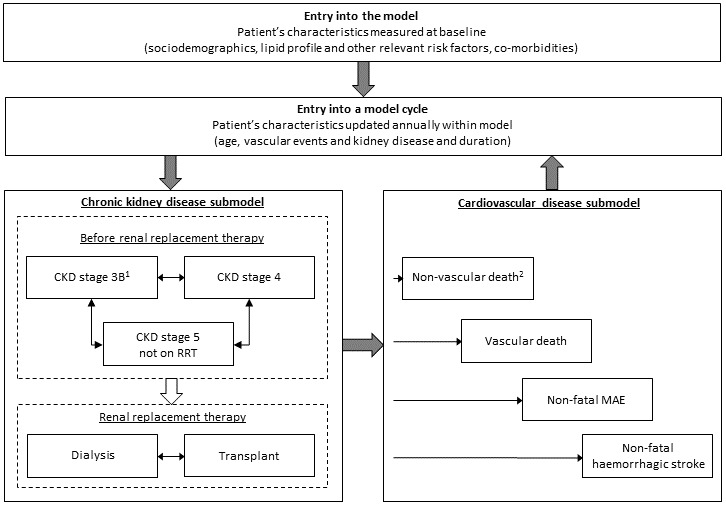
Schematic of the SHARP CKD-CVD lifetime health outcomes model. ^1^A small number of participants had estimated glomerular filtration rate ≥45 mL/min/1.73 m^2^ at entry into the study; ^2^Cox proportional hazards model derived from SHARP data used in internal validation. UK population data used to derive annual non-vascular mortality rates in the model. CKD, chronic kidney disease; CVD, cardiovascular disease; MAE, major atherosclerotic event; RRT, renal replacement therapy; SHARP, Study of Heart and Renal Protection.

The CVD and CKD submodels were combined into the *SHARP CKD-CVD model,* a Markov model with an annual cycle of transition (figure 1). A cohort simulation^12^ was used to evaluate health outcomes and costs for individual participants annually until death or 95 years of age. The model states were defined by the most recent event (non-vascular death, vascular death, non-fatal major atherosclerotic event or non-fatal haemorrhagic stroke; CVD submodel) and the most recent CKD status (CKD stage 3B, CKD stage 4, CKD stage 5 not on RRT, on dialysis, with kidney transplant; CKD submodel). Together, the model states comprise all possible combinations of non-fatal CVD (including no previous CVD) and CKD states, as well as two fatal states: vascular and non-vascular death. Parameter uncertainty was assessed using the bootstrap method^12^ with 1000 resamples of risk equations, and confidence intervals were derived using the equal-tailed percentile method.

### Model validation

Model-simulated cumulative rates of cardiovascular endpoints (all participants) and progression to RRT (those not on RRT at entry) were internally validated through comparison with the 5-year Kaplan-Meier product-limit estimates in SHARP, across all participants, and in subgroups by CKD status. Model discrimination was assessed using Harrell’s c-index for a censored response.^13^ In further validation, data from the Chronic Renal Impairment in Birmingham (CRIB), prospective observational study of 382 patients with CKD stage 3–5^14^ and two randomised trials in dialysis patients, the Der Deutsche Diabetes Dialyse Studie (4D) (1255 patients with type 2 diabetes mellitus receiving maintenance haemodialysis^15^) and A Study to Evaluate the Use of Rosuvastatin in Subjects on Regular Hemodialysis: An Assessment of Survival and Cardiovascular Events (AURORA) (2776 patients on maintenance haemodialysis^16^), were used. Kaplan-Meier curves and Harrell’s c-index were used to assess calibration and discrimination, respectively.

The SHARP CKD-CVD model was also assessed against recommended risk scores. First, the 5-year risks of a major vascular event or vascular death in participants without previous CVD or diabetes in SHARP and AURORA were calculated using (1) the Pooled Cohort Risk Equations^17 18^ and (2) the SHARP CKD-CVD model, and compared with observed rates. Second, the 5-year risk of progressing to RRT (ie, initiation of dialysis or renal transplantation) among SHARP participants not on dialysis at study entry, and among CRIB participants, was calculated using (1) the model by Tangri *et al* for progression of CKD to kidney failure^19^ and (2) the SHARP CKD-CVD model, and compared with the observed rates.

### Health-related quality of life

Of the 7018 SHARP participants alive at the final follow-up, 6356 completed the EuroQol 5-dimensions 3-level (EQ-5D-3L) questionnaire (including mobility, self-care, usual activities, pain/discomfort and anxiety/depression quality-of-life dimensions).^20^ The responses were used to calculate utilities using the UK EQ-5D-3L tariff,^21^ taking values between 1 (full health) and −0.596 (worse than death; 0 corresponds to death). Linear regression was used to model the effects of participant characteristics on quality of life and predict patient’s quality of life during each annual period in the model.

### Hospital care costs

Annual hospital care costs associated with model states, derived from SHARP,^22^ were implemented in the model.

## Results

In SHARP, 9270 participants with moderate-to-advanced CKD were followed for an average of 4.9 years (total 39 386 person-years).^9^ At study entry, mean age was 62 years (SD 12), 5800 participants (63%) were male, 5152 (56%) were recruited in Europe, 6646 (72%) were white, 2094 (23%) had diabetes and 1393 (15%) had prior vascular disease (see online supplementary table S1).

In total, 736 vascular deaths, 1753 major atherosclerotic events or vascular deaths and 1785 major vascular events or vascular deaths contributed to cardiovascular risks’ estimation (see online supplementary table S2). Of the 6235 participants not on RRT at entry, 1739 (28%; of which 913 were in CKD stage 5 not on RRT) initiated dialysis and 462 (7%; of which 255 were in CKD stage 5 not on RRT) received kidney transplants during the study. Of the 3025 participants on dialysis at entry, 669 received kidney transplants. Excluding transplants failing in the year of transplantation, the annual probability of transplant failure in SHARP in the model was 1.3% (see online supplementary table S3).

### Cardiovascular risk equations

Non-fatal cardiovascular events were strongly associated with an increased subsequent cardiovascular risk, with the increase greatest in the first year (table 1). Compared with participants without vascular disease, vascular death rates were three times higher (hazard ratios (HR)) 3.0 (95% CI 2.2 to 4.0)) in the year following a major atherosclerotic event, more than twice as high (HR 2.6 (1.7–3.9)) in the subsequent year and remained 50% higher (HR 1.5 (0.9–2.7)) thereafter. Following a non-fatal major atherosclerotic event, the HRs for another such event or death from a vascular cause in the first, second and subsequent years were 4.3 (3.6–5.2), 3.5 (2.7–4.6) and 2.3 (1.6–3.3), respectively.

**Table 1:** Multivariate risk equations in the SHARP CKD-CVD lifetime outcomes model

Covariates*	Category	CVD submodel†			CKD submodel				Probability of kidney transplant among those on dialysis in previous year
		Vascular death	MAE or vascular death	MVE or vascular death	From any pre-RRT stage‡				
					CKD stage 3B versus CKD stage 4	CKD stage 5, not RRT versus CKD stage 4	Renal transplant versus CKD stage 4	Dialysis versus CKD stage 4	
		Exponential PH HR (95% CI)	Gompertz PH HR (95% CI)	Gompertz PH HR (95% CI)	Multinomial logistic regression RRR^2^ (95% CI)				Logistic regression OR (95% CI)
**Patient’s characteristics at baseline**									
Sociodemographic characteristics									
Sex	Male	1.3 (1.1 to 1.6)	1.4 (1.2 to 1.5)	1.4 (1.2 to 1.5)	1.2 (1.1 to 1.4)	1.1 (1.0 to 1.2)	1.2 (0.9 to 1.7)	1.5 (1.3 to 1.7)	1.2 (1.0 to 1.4)
Ethnicity/country (ref: white)	Asian (China)	3.5 (2.4 to 5.0)	1.3 (1.0 to 1.7)	1.4 (1.1 to 1.8)	1.5 (1.2 to 1.9)	2.1 (1.7 to 2.7)	0.6 (0.2 to 1.9)	1.6 (1.2 to 2.2)	0.3 (0.2 to 0.6)
	Asian (other country)	1.2 (0.9 to 1.5)	0.7 (0.6 to 0.9)	0.7 (0.6 to 0.9)	0.9 (0.8 to 1.1)	1.1 (0.9 to 1.2)	0.1 (0.1 to 0.4)	0.9 (0.7 to 1.1)	0.1 (0.1 to 0.1)
	Black	0.9 (0.6 to 1.5)	0.8 (0.6 to 1.1)	0.9 (0.6 to 1.2)	1.2 (0.9 to 1.7)	1.1 (0.7 to 1.7)	1.0 (0.2 to 4.4)	1.9 (1.2 to 3.3)	0.5 (0.4 to 0.8)
	Other	1.2 (0.8 to 1.8)	0.7 (0.6 to 1.0)	0.7 (0.6 to 1.0)	1.0 (0.7 to 1.5)	1.2 (0.9 to 1.7)	2.7 (1.4 to 5.1)	1.4 (0.9 to 2.1)	0.6 (0.4 to 0.8)
Smoker (ref: never)	Former	1.0 (0.8 to 1.2)	1.1 (1.0 to 1.2)	1.1 (1.0 to 1.2)					1.0 (0.8 to 1.1)
	Current	1.4 (1.1 to 1.7)	1.4 (1.2 to 1.6)	1.4 (1.2 to 1.6)					0.7 (0.6 to 0.9)
Adult dependants (ref: yes)	No								0.7 (0.6 to 0.8)
	Missing								0.8 (0.6 to 1.0)
Education level (ref: A-levels or above)	GCSE/vocational				1.0 (0.9 to 1.2)	1.1 (0.9 to 1.2)	0.7 (0.5 to 1.0)	1.2 (1.1 to 1.4)	0.6 (0.5 to 0.8)
	Below secondary				1.0 (0.9 to 1.2)	0.8 (0.7 to 1.0)	0.4 (0.2 to 0.7)	0.9 (0.7 to 1.0)	0.8 (0.6 to 1.0)
					1.0 (0.8 to 1.1)	1.1 (0.9 to 1.3)	0.8 (0.5 to 1.2)	1.1 (0.9 to 1.3)	1.0 (0.8 to 1.2)
BMI, kg/m^2^ (ref: ≥25, <30)	<25								1.0 (0.9 to 1.2)
	≥30								0.7 (0.6 to 0.9)
Disease history, laboratory measurements and other risk factors									
Diabetes	Yes	1.4 (1.1 to 1.8)	1.4 (1.2 to 1.7)	1.4 (1.2 to 1.6)	1.0 (0.8 to 1.2)	0.9 (0.7 to 1.0)	0.2 (0.1 to 1.0)	1.2 (0.9 to 1.5)	0.6 (0.4 to 0.9)
Previous failed kidney transplant	Yes								0.5 (0.4 to 0.7)
Diastolic blood pressure, mm Hg (ref: ≥75, <85)	<75								0.8 (0.6 to 0.9)
	≥85								1.1 (0.9 to 1.3)
Systolic blood pressure, mm Hg (ref: ≥130, <150)	<130	1.2 (1.0 to 1.4)	1.0 (0.9 to 1.2)	1.0 (0.9 to 1.2)					1.0 (0.9 to 1.2)
	≥150	1.3 (1.1 to 1.6)	1.2 (1.1 to 1.3)	1.2 (1.1 to 1.4)					0.8 (0.6 to 0.9)
Albumin, g/dL (ref ≥3.9, <4.2)	<3.9	1.3 (1.1 to 1.5)	1.2 (1.1 to 1.4)	1.2 (1.1 to 1.4)	0.9 (0.8 to 1.0)	1.1 (1.0 to 1.3)	1.4 (0.9 to 2.2)	1.4 (1.2 to 1.7)	
	≥4.2	0.8 (0.6 to 0.9)	0.9 (0.8 to 1.0)	0.9 (0.8 to 1.0)	1.0 (0.9 to 1.1)	1.0 (0.9 to 1.1)	1.1 (0.7 to 1.5)	0.9 (0.8 to 1.1)	
	Missing	1.1 (0.8 to 1.5)	1.1 (0.9 to 1.3)	1.1 (0.9 to 1.3)	0.7 (0.6 to 0.9)	1.1 (0.9 to 1.3)	1.5 (0.8 to 2.8)	1.4 (1.1 to 1.8)	
Haemoglobin, g/dL (ref: ≥11.6, <13)	<11.6	1.5 (1.2 to 1.8)	1.3 (1.1 to 1.4)	1.3 (1.1 to 1.4)	1.0 (0.9 to 1.2)	1.2 (1.0 to 1.3)	1.0 (0.7 to 1.5)	1.3 (1.1 to 1.6)	
	≥13	0.9 (0.7 to 1.2)	1.0 (0.9 to 1.2)	1.0 (0.9 to 1.1)	1.3 (1.2 to 1.5)	0.8 (0.7 to 0.9)	0.6 (0.4 to 0.8)	0.7 (0.6 to 0.8)	
	Missing	1.0 (0.7 to 1.5)	1.1 (0.9 to 1.4)	1.1 (0.9 to 1.4)	1.1 (0.9 to 1.3)	1.0 (0.8 to 1.3)	1.0 (0.4 to 2.9)	1.1 (0.8 to 1.6)	
Phosphate, mmol/L (ref: ≥1.2, <1.5)	<1.2				1.3 (1.1 to 1.4)	0.7 (0.6 to 0.7)	0.9 (0.6 to 1.3)	0.6 (0.5 to 0.8)	
	≥1.5				0.8 (0.6 to 1.0)	1.4 (1.2 to 1.6)	1.3 (0.9 to 1.9)	1.7 (1.5 to 2.1)	
	Missing				1.5 (1.3 to 1.8)	0.7 (0.6 to 0.9)	0.5 (0.2 to 1.0)	0.9 (0.7 to 1.1)	
Urinary ACR (measured in pre-RRT participants only), mg/g (ref: <30)	≥30, ≤300	1.0 (0.7 to 1.4)	1.2 (1.0 to 1.5)	1.2 (1.0 to 1.5)	0.6 (0.5 to 0.7)	1.7 (1.5 to 2.0)	0.9 (0.5 to 1.5)	1.7 (1.3 to 2.1)	
	>300	1.3 (0.9 to 1.8)	1.4 (1.1 to 1.7)	1.4 (1.1 to 1.7)	0.3 (0.3 to 0.4)	3.0 (2.5 to 3.5)	2.8 (1.7 to 4.8)	4.9 (3.8 to 6.3)	
	Missing	1.2 (0.8 to 1.9)	1.0 (0.8 to 1.4)	1.1 (0.8 to 1.4)	0.9 (0.8 to 1.1)	1.6 (1.3 to 2.0)	1.1 (0.5 to 2.5)	1.9 (1.4 to 2.7)	
	RRT	1.2 (0.8 to 1.8)	1.3 (1.0 to 1.8)	1.4 (1.0 to 1.8)					
HDL cholesterol, mmol/L (ref: ≥0.9, <1.2)	<0.9				1.0 (0.9 to 1.1)	1.1 (0.9 to 1.2)	0.8 (0.5 to 1.2)	1.1 (0.9 to 1.3)	
	≥1.2				1.1 (1.0 to 1.3)	0.8 (0.7 to 0.9)	0.9 (0.6 to 1.3)	0.9 (0.8 to 1.1)	
Cause of kidney disease (ref: other/unknown)	Diabetic nephropathy	1.3 (1.0 to 1.7)	1.4 (1.2 to 1.6)	1.4 (1.2 to 1.6)	0.8 (0.7 to 1.0)	0.9 (0.7 to 1.1)	2.1 (0.4 to 10.6)	1.1 (0.8 to 1.5)	0.8 (0.5 to 1.3)
	Cystic kidney disease	0.8 (0.5 to 1.0)	0.9 (0.7 to 1.0)	0.9 (0.7 to 1.1)	0.3 (0.3 to 0.4)	2.6 (2.3 to 3.0)	4.9 (3.5 to 6.9)	4.9 (4.1 to 5.9)	1.6 (1.3 to 1.8)
**Characteristics updated on an annual basis**									
Age	Per 10 years older	1.6 (1.5 to 1.7)	1.4 (1.4 to 1.5)	1.4 (1.3 to 1.5)	0.8 (0.8 to 0.9)	0.9 (0.9 to 1.0)	0.4 (0.4 to 0.5)	0.8 (0.7 to 0.8)	0.6 (0.6 to 0.6)
Time since CKD diagnosis	Per 10 years				1.0 (0.9 to 1.0)	1.1 (1.0 to 1.1)	1.2 (1.1 to 1.3)	1.1 (1.0 to 1.1)	
Previous (most recent) CVD event (ref: no MVE within trial, no baseline vascular disease)	No MVE within trial, with baseline vascular disease	1.7 (1.4 to 2.1)	1.9 (1.7 to 2.2)	1.9 (1.7 to 2.2)	1.0 (0.9 to 1.2)	1.0 (0.9 to 1.2)	0.6 (0.3 to 1.2)	1.0 (0.8 to 1.2)	0.7 (0.5 to 0.9)
	MAE within trial, last year	3.0 (2.2 to 4.0)	4.3 (3.6 to 5.2)	4.3 (3.5 to 5.1)	NA	NA			
	MAE within trial, 1–2 years ago	2.6 (1.7 to 3.9)	3.5 (2.7 to 4.6)	3.5 (2.7 to 4.5)					
	MAE within trial, >2 years ago	1.5 (0.9 to 2.7)	2.3 (1.6 to 3.3)	2.3 (1.6 to 3.2)					
	No MAE, but MVE within trial	4.6 (1.7 to 12.5)	3.4 (1.4 to 8.2)	4.3 (1.8 to 10.0)					
	MVE within trial§	NA			0.9 (0.7 to 1.2)	1.6 (1.2 to 2.1)	1.3 (0.3 to 5.1)	3.0 (2.1 to 4.2)	0.9 (0.7 to 1.3)
CKD status at the end of the previous cycle (ref: CKD stage 3B)	CKD stage 4	1.7 (1.3 to 2.3)	1.3 (1.1 to 1.5)	1.3 (1.1 to 1.5)	0.02 (0.02 to 0.02)	10.8 (6.8 to 17.0)	6.5 (0.9 to 48.2)	2.6 (1.6 to 4.0)	NA
	CKD stage 5, not on RRT	2.2 (1.6 to 3.1)	1.6 (1.3 to 2.0)	1.6 (1.3 to 1.9)	0.01 (0.01 to 0.02)	737.1 (455.7 to 1192.4)	1279.5 (174.2 to 9397.7)	369.4 (233.5 to 584.3)	
	On dialysis, RRT≤3 years	2.8 (2.0 to 4.0)	2.1 (1.7 to 2.7)	2.1 (1.7 to 2.6)	NA				
	On dialysis, RRT>3 years	4.1 (2.7 to 6.1)	2.5 (1.9 to 3.3)	2.5 (1.9 to 3.3)				1.2 (1.1 to 1.4)¶	
	Functioning kidney transplant	0.9 (0.4 to 2.0)	0.7 (0.5 to 1.2)	0.8 (0.5 to 1.2)				NA	
Intercept**		−14.8 (−15.5 to -14.1)	−12.4 (−12.8 to -12.0)	−12.4 (−12.8 to -11.9)	9.3 (6.6 to 13.0)	0.0 (0.0 to 0.0)	0.1 (0.0 to 0.5)	0.0 (0.0 to 0.0)	3.0 (1.9 to 5.0)
Ancillary parameter (on the original scale)		NA	−0.0002 (−0.0003, −0.0001)	−0.0002 (−0.0003, −0.0001)	NA				NA

*Missing data were either assigned into a separate category or, if data were missing for fewer than 5% of participants, to the middle group (online supplementary item S1 and table SM1), †Each risk equation included an adjustment for use of lipid-lowering therapy, ‡The coefficients represent the RRR for being in the respective CKD category versus the comparator category (CKD stage 4) per unit change in the covariate. For example, the RR of being in stage 3B relative to being in stage 4 in males is 1.2 times higher than in females. To calculate RR relative to another stage category, the corresponding RRRs need to be divided. For example, the RR of being on kidney transplant relative to being in CKD stage 5 in males is 1.2 (=1.3/1.1) times higher in males than in females; years in which deaths occurred were excluded from modelling, §In the CKD risk equations, all within-trial major vascular events were combined into one category, ¶Reference category is ‘on dialysis’, RRT≤3 years, **Intercept in the CVD submodel is presented on the original scale.

ACR, albumin:creatinine ratio; BMI, body mass index; CKD, chronic kidney disease; CVD, cardiovascular disease; MAE, major atherosclerotic event; MVE, major vascular event; NA, not applicable: covariate not included or specified through other covariates within category; PH, proportional hazards; RRR, relative risk ratio; RRT, renal replacement therapy.

More advanced CKD status was also associated with increased cardiovascular risk (table 1). After adjustment for covariates, compared with participants in CKD stage 3B, the risk of dying from vascular causes in the exponential proportional hazards survival model was about twice as high in participants in stage 4 or stage 5 not on RRT (HR 1.7 (1.3–2.3) and HR 2.2 (1.6–3.1), respectively) and three to four times higher in those on dialysis (HR 2.8 (2.0–4.0) for participants with RRT duration <3 years and HR 4.1 (2.7–6.1) for those with RRT duration ≥3 years). A graded association was also observed for major atherosclerotic event or vascular death risk in the Gompertz proportional hazards model (compared with CKD stage 3B, HR 1.3 (1.1–1.5) for participants in stage 4, HR 1.6 (1.3–2.0) for participants in stage 5, HR 2.1 (1.7–2.7) for participants on dialysis with RRT <3 years and HR 2.5 (1.9–3.3) for participants on dialysis with RRT ≥3 years). Participants in receipt of RRT at entry were at further increased cardiovascular risk conferred through the hazard for the separate RRT category in the uACR covariate (table 1). Cardiovascular risks in participants with kidney transplants were similar to those in CKD stage 3B (HR 0.9 (0.4–2.0) for risk of vascular death and HR 0.7 (0.5–1.2) for risk of major atherosclerotic event or vascular death).

### CKD risk equations

The main predictor of CKD progression was previous CKD stage (table 1). Compared with participants in CKD stage 3B, participants in stage 4 were almost 11 times more likely to progress to stage 5 (relative risk ratio 10.8 (95%CI 6.8 to 17.0)) and 2.6 times (1.6–4.0) more likely to start dialysis the following year than to remain in stage 4. Similarly, among participants in CKD stage 4, compared with participants with baseline uACR <30 mg/g, those with baseline uACR of 30–300 mg/g were 1.7 times (1.5–2.0) more likely to progress to stage 5 and 1.7 times (1.3–2.1) more likely to start dialysis the following year than to remain in stage 4.

### Effects of CVD events and CKD progression on health-related quality of life

Cardiovascular events and on dialysis status were the main determinants of quality of life (table 2). Major vascular events were associated with reductions in EQ-5D utility of 0.17 (95%CI 0.14 to 0.21) in the year of event and 0.10 (0.08–0.13) in subsequent years, while on dialysis status was associated with a reduction of 0.06 (0.04–0.07).

**Table 2:** Health-related quality of life in moderate-to-advanced chronic kidney disease: a linear regression model derived from Study of Heart and Renal Protection data

Participant’s characteristics		Effect on quality of life, as measured by EQ-5D utility (95% CI)
Intercept*		0.86 (0.84 to 0.88)
Sex	Male	0.06 (0.05 to 0.07)
Ethnicity/country (ref: white)	Asian (China)	0.02 (−0.01 to 0.04)
	Asian (other country)	0.06 (0.04 to 0.08)
	Black	−0.04 (−0.08 to −0.01)
	Other	−0.02 (−0.06 to 0.01)
Smoker (ref: never)	Former	−0.01 (−0.02 to 0.00)
	Current	−0.04 (−0.06 to −0.02)
Education level (ref: A-levels or above)	Secondary/vocational	−0.02 (−0.03 to 0.00)
	Below secondary	−0.04 (−0.05 to −0.02)
	Missing	−0.03 (−0.05 to −0.01)
BMI, kg/m^2^ (ref:≥25,<30)	<25	0.01 (0.00 to 0.02)
	≥30	−0.04 (−0.06 to −0.03)
Previous failed kidney transplant	Yes	−0.07 (−0.11 to −0.03)
Diabetic nephropathy	Yes	−0.06 (−0.08 to −0.04)
**Participant’s characteristics during the study**		
Age (centred at 60 years)	Per 10 years older	−0.05 (−0.05 to −0.04)
Previous (most recent) MVE (ref: no MVE within trial, no baseline vascular disease)	No MVE within trial, with baseline vascular disease	−0.07 (−0.09 to −0.05)
	MVE within trial, last year	−0.17 (−0.21 to −0.14)
	MVE within trial,>1 years ago	−0.10 (−0.13 to −0.08)
Being on dialysis	Yes	−0.06 (−0.07 to −0.04)

*The intercept term corresponds to the quality of life (ie, EQ-5D utility) of a 60-year-old white female with CKD but not on dialysis, non-smoker, with education level of A-levels or above (or equivalent), BMI ≥25 <30 kg/m^2^, without previously failed transplant, without diabetic nephropathy and without history of vascular disease.

BMI, body mass index; MVE, major vascular event.

### Model validation

**Figure 2:**
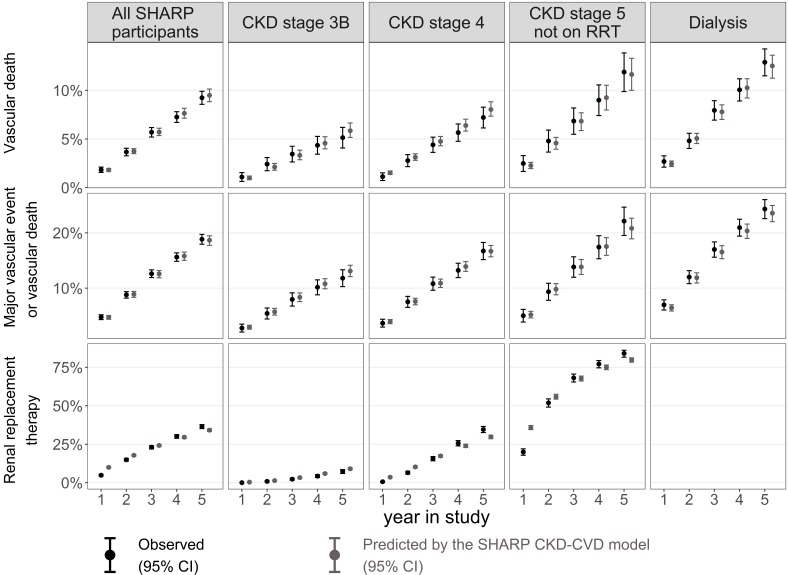
Predicted and observed Kaplan-Meier cumulative risks in SHARP (internal validity). For the progression to renal replacement therapy (RRT) endpoint, only participants in pre-RRT stages at baseline were included. Lipid-lowering treatment use was simulated as the percentage of participants using any lipid-lowering medication in the respective treatment arm and year of follow-up in SHARP. CKD, chronic kidney disease; CVD, cardiovascular disease; SHARP, Study of Heart and Renal Protection.

The cumulative event rates predicted by the SHARP CKD-CVD model closely matched observed rates for almost all years of follow-up and all participant subgroups (figure 2), and Harrell’s c-index showed good discrimination for all disease endpoints (see online supplementary table S4).

**Figure 3:**
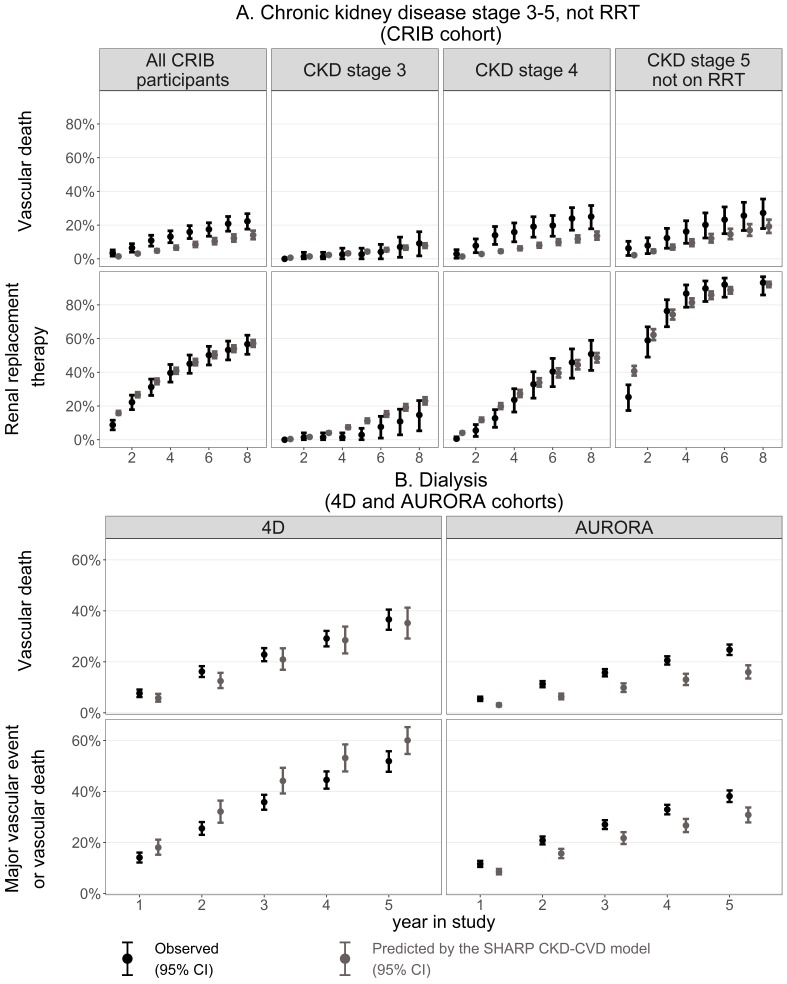
Predicted and observed Kaplan-Meier cumulative risks in CRIB, 4D and AURORA (external validity). The predictions were simulated in the absence of study lipid-lowering treatments in 4D and AURORA; information on non-coronary revascularisation was not available in 4D. Transplantation rates calibrated to correspond to rates in 4D and AURORA; no other calibration was performed. AURORA, A Study to Evaluate the Use of Rosuvastatin in Subjects on Regular Hemodialysis: An Assessment of Survival and Cardiovascular Events; CKD, chronic kidney disease; CRIB, Chronic Renal Impairment in Birmingham; CVD, cardiovascular disease; RRT, renal replacement therapy; 4D, Der Deutsche Diabetes Dialyse.

Participants in CRIB, 4D and AURORA differed from participants in SHARP (see online supplementary table S5), being more likely to have had vascular disease at entry. Also, while participants on dialysis at entry into SHARP had been on dialysis for an average of 2.5 (SD 3.1) years, those in 4D had received dialysis for an average of 0.7 (0.6) years and those in AURORA for 4.4 (5.2) years. In the predialysis CRIB cohort, SHARP CKD-CVD model predictions showed good agreement with observed risks of vascular death and progression to RRT in most subgroups and years (figure 3). In the 4D dialysis cohort, the agreement between predicted and observed risks was good for vascular death but a 14% higher rate was predicted for the combined endpoint by year 5 (figure 3). In the AURORA dialysis cohort, the model captured the trends in events over time but the predicted rates of major vascular events or vascular deaths and of vascular death alone were 19% and 35% lower, respectively, by year 5 (figure 3). In these external cohorts, Harrell’s c-index indicates that the model discriminates risk well in predialysis (c-index in CRIB 0.75 to 0.84) but its ability to discriminate risk in dialysis might be more limited (c-index 0.65 in AURORA; 0.58 to 0.61 in 4D; see online supplementary table S4).

The cardiovascular risk predictions by the Pooled Cohort Equations could not capture variation in risk across CKD stages, unlike predictions from the SHARP CKD-CVD model, with deviations particularly pronounced among SHARP participants in CKD stage 3B and those on dialysis (table 3). The 5-year rates of progression to RRT predicted by the SHARP CKD-CVD and the Tangri models were both close to the observed rates among pre-RRT SHARP and CRIB participants.

**Table 3:** Comparison of predictions of 5-year risk of a major vascular event or vascular death and initiation of RRT by the SHARP CKD-CVD model and external risk models

A. 5-year risk (%) of a major vascular event or vascular death*			
	Observed (95% CI)	Predicted by the SHARP CKD-CVD model	Predicted by the Pooled Cohort Equations
SHARP participants overall	14.3 (12.8 to 15.7)	13.8	15.2
Stage 3B	8.6 (6.2 to 11.0)	9.4	15.3
Stage 4	12.7 (10.2 to 15.1)	12.3	15.7
Stage 5, not on RRT	16.4 (12.4 to 20.2)	15.6	20.5
On dialysis	19.3 (16.2 to 22.2)	17.9	12.4
AURORA participants; on dialysis	26.3 (23.0 to 30.0)	20.1	9.4

B. 5-year risk (%) of initiation of RRT			
	Observed (95% CI)	Predicted by the SHARP CKD-CVD model	Predicted by the Tangri et al model
SHARP participants overall	36.5 (35.2 to 37.8)	34.2	34.3
Stage 3B	7.3 (6.0 to 8.5)	9.1	7.7
Stage 4	34.6 (32.6 to 36.6)	29.8	32.0
Stage 5, not on RRT	84.0 (81.6 to 86.1)	79.9	76.0
CRIB participants overall	45.1 (39.4 to 50.2)	46.0	49.4

*Participants with baseline vascular disease, diabetes and low-density lipoprotein (LDL) cholesterol <70 or >189 mg/dL, as well as ezetimibe/simvastatin-allocated participants in SHARP were excluded from the analyses. The Pooled Cohort Equations predict the risk of the first atherosclerotic cardiovascular disease (ASCVD) event, while the first major vascular event or vascular death endpoint in SHARP and AURORA also include revascularisations and non-ASCVD vascular deaths. Therefore, the risks produced by the Pooled Cohort Equations were calibrated by a factor corresponding to the proportion of revascularisations and non-ASCVD vascular deaths in the respective group of participants.

AURORA, A Study to Evaluate the Use of Rosuvastatin in Subjects on Regular Hemodialysis: An Assessment of Survival and Cardiovascular Events; CKD, chronic kidney disease; CRIB, Chronic Renal Impairment in Birmingham; CVD, cardiovascular disease; LDL, low density lipoprotein; RRT, renal replacement therapy; SHARP, Study of Heart and Renal Protection.

We illustrate the use of the model in two applications.

### Application 1: prediction of long-term outcomes in CKD

**Figure 4:**
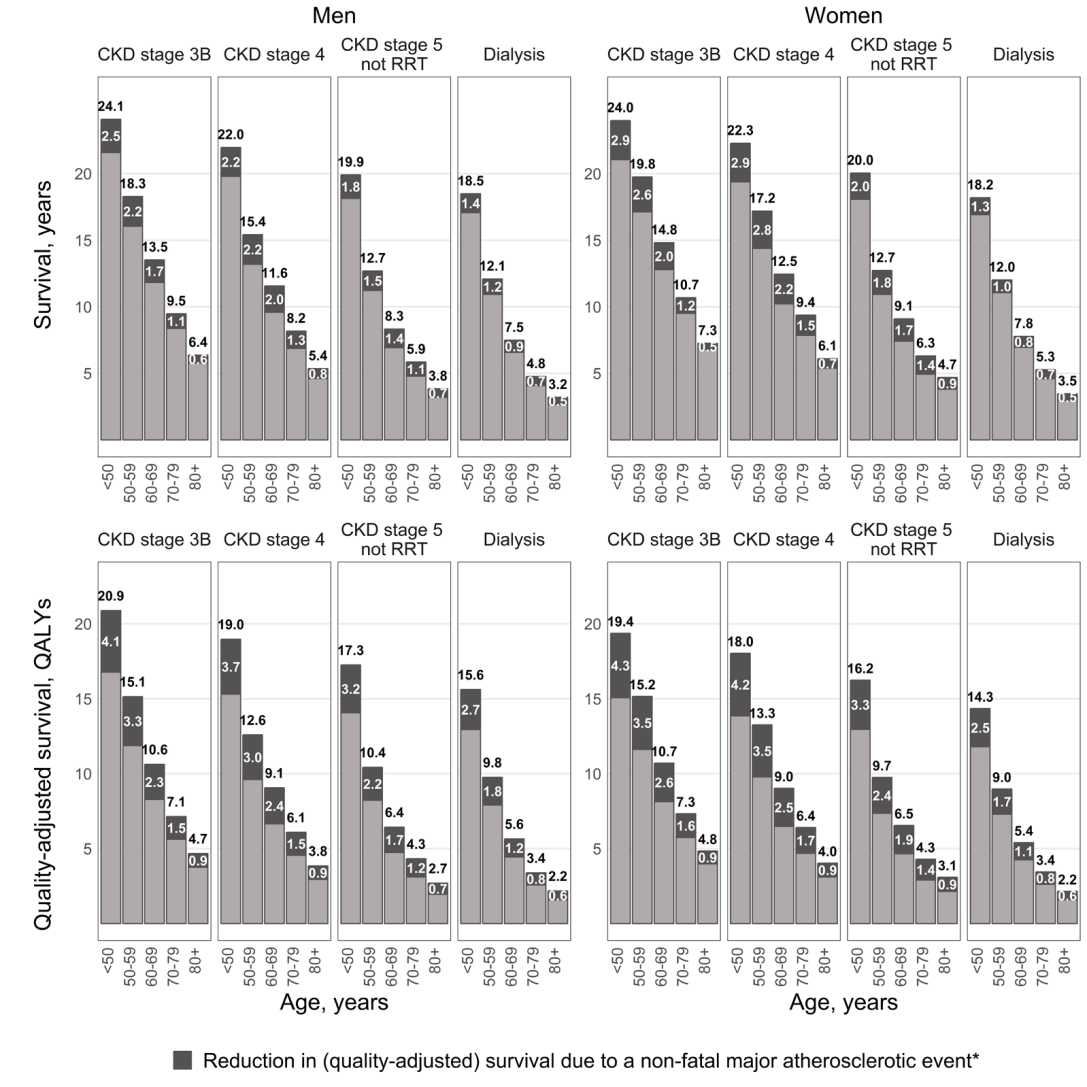
Predicted life expectancy (years) and quality-adjusted life-years for participants in SHARP. The predictions are for SHARP participants in the absence of lipid-lowering treatment. *Major atherosclerotic event is assumed to occur in year prior to entry in the model. CKD, chronic kidney disease; QALYs, quality-adjusted life years; RRT, renal replacement therapy; SHARP, Study of Heart and Renal Protection.

The CKD-CVD model was used to project survival for SHARP participants using their baseline characteristics. The model predicted marked variation in survival by CKD stage (figure 4, online supplementary table S6). Among participants aged 60–69 years in CKD stage 3B, men were predicted to survive a further 13.5 (95%CI 13.2 to 13.9) years (10.6 (10.3–10.9) quality-adjusted life years (QALYs)) and women 14.8 (14.4–15.3) years (10.7 (10.3–11.1) QALYs). However, corresponding predictions for those on dialysis were only 7.5 (7.2–7.8) years (5.6 (5.4–5.9) QALYs) and 7.8 (7.5–8.1) years (5.4 (5.2–5.6) QALYs). At ages 60–69, a major, but non-fatal, atherosclerotic event was predicted to reduce life expectancy by 1.7 (0.8–2.5) years (2.3 (1.6–3.0) QALYs) for a man in CKD stage 3B and 2.0 (1.0–2.9) years (2.6 (1.8–3.2) QALYs) for a woman in CKD stage 3B. Corresponding results for those on dialysis were 0.9 (0.3–1.4) years (1.2 (0.8–1.6) QALYs) and 0.8 (0.3–1.4) years (1.1 (0.7–1.5) QALYs).

### Application 2: prediction of risk in individual patients with CKD

The SHARP CKD-CVD model can predict disease risks in people with CKD given their individual demographic and clinical characteristics. For example, the 5-year major vascular event risk for a patient with stage 4 CKD is 7% (95%CI 5% to 9%) for a 50-year-old male with cystic kidney disease but no other cardiovascular risk factors or comorbidities, and 49% (40%–59%) for a 70-year-old male with diabetic nephropathy and prior CVD (table 4). Similarly, the predicted 5-year death risk is 33% (31%–34%) for a 60-year female without major comorbidity at dialysis initiation, but is only 13% (13%–14%) if she is transplanted.

**Table 4:** An illustrative model application: 5-year risk of adverse outcomes in selected patients

Patient profile (see footnote for complete profiles)	Major vascular event or vascular death	Renal replacement therapy	Vascular death	Death from any cause*
Heterogeneity among patients in the same CKD stage				
Fifty-year-old male in CKD stage 4, with cystic kidney disease, and no comorbidities†	7% (5%–9%)	65% (58%–72%)	2% (1%–2%)	9% (8%–10%)
Seventy-year-old male in CKD stage 4, with diabetic nephropathy and cardiovascular disease‡	49% (40%–59%)	14% (11%–18%)	15% (10%–23%)	27% (23%–34%)
Initiation of renal replacement therapy: comparison of outcomes for patients on dialysis versus those on kidney transplant				
Sixty-year-old female without comorbidities following initiation of dialysis§	12% (10%–14%)	NA	4% (3%–6%)	33% (31%–34%)
Sixty-year-old female without comorbidities following successful kidney transplant¶	5% (3%–7%)	NA	1% (1%–2%)	13% (13%–14%)

*UK population data used to derive annual non-vascular mortality rates in the model (Table SM2), †Patient profiles: 50 years old; male; white; postsecondary education; adult dependants; never smoker; BMI: 25–29 kg/m^2^; diastolic blood pressure≥85 mm Hg; systolic blood pressure<130 mm Hg; HDL cholesterol<0.9 mmol/L; albumin: 3.9–4.1 g/dL; haemoglobin≥13 g/dL; phosphate 1.2–1.4 mmol/L; uACR: 30–300 mg/g; no vascular disease; no diabetes mellitus; CKD stage 4; 30 years since CKD diagnosis; cystic kidney disease, ‡Seventy years old; male; white; completed secondary education; no adult dependants; current smoker; BMI≥30 kg/m^2^; diastolic blood pressure<75 mm Hg; systolic blood pressure:<130 mm Hg; HDL cholesterol<0.9 mmol/L; albumin≥4.2 g/dL; haemoglobin≥13 g/dL; phosphate<1.2 mmol/L; ACR: 30–300 mg/g; vascular disease; diabetes mellitus; CKD stage 4; 20 years since CKD diagnosis; diabetic nephropathy, §Sixty years old; female; white; completed secondary education; adult dependants; never smoker; BMI<25 kg/m^2^; diastolic blood pressure: 75–84 mm Hg; systolic blood pressure: 130–149 mm Hg; HDL cholesterol: 0.9–1.1 mmol/L; albumin: 3.9–4.1 g/dL; haemoglobin: 11.6–12.9 g/dL; phosphate≥1.5 mmol/L; no previous vascular disease; no diabetes mellitus; initiating dialysis; 5 years since CKD diagnosis; no cystic kidney disease or diabetic nephropathy, ¶Same as profile 3 but following successful kidney transplant.

CKD, chronic kidney disease; RRT, renal replacement therapy; BMI, body mass index; uACR, urinary albumin-to-creatinine ratio.

### Model web interface

The SHARP CKD-CVD model interface and user guide are available at http://dismod.ndph.ox.ac.uk/kidneymodel/app/.

## Discussion

The SHARP CKD-CVD model, developed using the detailed data from the 9270 participants with moderate-to-advanced CKD in SHARP, projects long-term cardiovascular event risk, kidney disease progression and (quality-of-life adjusted) survival. The estimated graded associations between more advanced CKD and higher cardiovascular risk and subsequent adverse renal and cardiovascular outcomes are consistent with published data.^2 23 24^ The model’s discrimination of risk was very good in participant categories in SHARP and predialysis CRIB cohorts, good in the dialysis AURORA cohort and informative, though more limited, in the dialysis 4D cohort with differences potentially due to the more homogeneous populations in AURORA and 4D (eg, 4D participants were with diabetes and both 4D and AURORA participants had substantially more cardiovascular comorbidities at entry). The ability of the model to discriminate risk reasonably well across moderate-to-advanced CKD categories suggests that the risk equations capture the important risk factors. The model’s ability to predict risks in external cohorts showed good performance in CRIB and 4D, but less consistent performance in AURORA, perhaps due to differences in cardiovascular comorbidity and the substantially longer duration spent on dialysis by AURORA patients at entry into the study. The model showed better ability to predict cardiovascular risk in SHARP participants than that of the Pooled Cohort Equations,^17 18^ and its ability to predict progression to RRT was comparable to that of the Tangri *et al* model.^19^

By contrast with the previously published models,^5–7^ the SHARP CKD-CVD model was derived entirely using the individual participant data from a large CKD cohort with adjudicated endpoints. The model takes account of the interdependence between kidney and cardiovascular diseases, includes separate kidney transplant and dialysis states and accounts for the effects of uncertainty in all model parameters on predictions. The freely available model interface allows adaptations to other settings, as well as simulation of patient outcomes with different cardiovascular interventions.

Potential limitations should be acknowledged. First, SHARP participants were followed for an average of 5 years and hence the longer term predictions are guided by the model structure and parametric proportional hazards assumptions. Nevertheless, the annual updating of age, CVD history and CKD status captures well the changes in hazard over time; the proportional hazards assumptions were satisfied in the survival models and supported in external validations; and the parameter uncertainty was fully incorporated. Second, SHARP excluded patients with major coronary disease, whereas in routine clinical practice coronary heart disease is highly prevalent in moderate-to-advanced CKD.^25^ While the inclusion of incident coronary events as time-updated covariates in risk equations enables the prediction of risks for kidney patients with coronary disease, model assessment in further CKD cohorts is desirable. Finally, future model developments could consider further disease markers and endpoints (eg, heart failure and sudden cardiac death,^26^ fractures, infections or cancers^27 28^).

The SHARP CKD-CVD model contributes to a greater understanding of risks of cardiovascular complications in CKD and will be useful to clinicians, analysts and policymakers to simulate long-term outcomes for their patients with CKD and evaluate the comparative effectiveness and cost-effectiveness of interventions to reduce cardiovascular risk.

**Key messages**

**What is already known on this subject?**

Chronic kidney disease (CKD) increases the risk of cardiovascular disease (CVD) and, conversely, CVD may affect kidney disease progression. Policy models to project the long-term health outcomes in people with CKD accounting for this interdependence, as well as for individual patients’ risk profiles, are unavailable.

**What does the study add?**

The Study of Heart and Renal Protection (SHARP) CKD-CVD model, developed using detailed data from nearly 10 000 patients with moderate-to-advanced CKD followed for an average of 5 years, projects lifetime cardiovascular event risks, kidney disease progression and (quality-of-life adjusted) survival. The model performs well in categories of patients by CKD stage in SHARP and in external CKD cohorts. A flexible user-friendly web interface, which also includes projection of healthcare costs, is freely available to facilitate model use.

**How might this impact on clinical practice?**

The model can be used by clinicians to evaluate long-term risks for their patients and discuss possible treatment options, as well as by analysts and policy makers to evaluate the comparative effectiveness and cost-effectiveness of interventions to manage cardiovascular complications in CKD.
