# Supplementary material for: A policy model of cardiovascular disease in moderate-to-advanced chronic kidney disease
Source: Heart. 2017 Aug 5;103(23):1880–90. doi: 10.1136/heartjnl-2016-310970 (PMC5749372; doi:10.1136/heartjnl-2016-310970)
Supplement: Supplementary material 1 [file heartjnl-2016-310970supp001.pdf]

Supplementary Material to "A policy model of cardiovascular disease in moderate-to-advanced chronic kidney disease"

Content **Page**

Table S1 Baseline characteristics of the 9270 SHARP participants, overall and by CKD status at baseline..2

Table S2 Cardiovascular disease events in SHARP contributing to cardiovascular risk estimation..4

Table S3 Transitions between CKD stages during the 41324 annual periods of follow-up in SHARP ………..5

Table S4 Harrell's C-index assessing discrimination of the SHARP CKD-CVD...................6

Table S5 Baseline characteristics of participants in CRIB, 4D and AURORA…………...........7

Table S6 Predicted life expectancy and quality-adjusted life years for participants in SHARP, by CKD stage at randomisation...9

Item S1 Supplementary methods...11

Table SM1 Missing biomarkers data at entry into SHARP …...11

Table SM2 Annual probabilities of non-vascular death in SHARP CKD-CVD model….......19

Table S1 Baseline characteristics of the 9270 SHARP participants, overall and by CKD status at baseline

	Overall	By CKD status at baseline[2]			
		Stage 3B[1] (eGFR≥30 to 45)[2]	Stage 4 (eGFR≥15 to <30)[2]	Stage 5, not on RRT (eGFR<15)[2]	On dialysis
	n = 9270	n = 2020	n = 2767	n = 1448	n = 3025
Age, years	62 (12)	62 (11)	64 (12)	62 (12)	60 (12)
Male	5800 (63%)	1461 (72%)	1653 (60%)	760 (52%)	1918 (63%)
Ethnicity/Country					
White	6646 (72%)	1389 (69%)	2124 (77%)	960 (66%)	2163 (72%)
Asian (China)	974 (11%)	338 (17%)	281 (10%)	311 (21%)	44 (1%)
Asian (other country)	1112 (12%)	193 (10%)	267 (10%)	132 (9%)	520 (17%)
Black	264 (3%)	62 (3%)	37 (1%)	20 (1%)	145 (5%)
Other	274 (3%)	38 (2%)	58 (2%)	25 (2%)	153 (5%)
Smoking status					
Current smoker	1243 (13%)	271 (13%)	336 (12%)	162 (11%)	472 (16%)
Ex-smoker	3272 (35%)	723 (36%)	1033 (37%)	480 (33%)	1033 (34%)
Alcohol drinker	2479 (27%)	674 (33%)	838 (30%)	352 (24%)	614 (20%)
Highest level of education					
A-levels or above	2333 (25%)	606 (30%)	708 (26%)	353 (24%)	660 (22%)
Secondary/vocational	3881 (42%)	833 (41%)	1140 (41%)	610 (42%)	1296 (43%)
Below secondary	1725 (19%)	322 (16%)	509 (18%)	298 (21%)	595 (20%)
No information	1331 (14%)	259 (13%)	410 (15%)	187 (13%)	474 (16%)

Any adult dependants	5247 (57%)	1212 (60%)	1601 (58%)	800 (55%)	1628 (54%)
Previous Vascular Disease	1393 (15%)	283 (14%)	430 (16%)	217 (15%)	461 (15%)
Diabetes Mellitus	2094 (23%)	469 (23%)	662 (24%)	293 (20%)	668 (22%)
Previous failed kidney transplant	239 (3%)	0 (0%)	0 (0%)	0 (0%)	236 (8%)
Body-Mass Index, kg/m²	27 (6)	28 (5)	28 (6)	27 (5)	26 (6)
Diastolic blood pressure, mmHg	79 (13)	80 (13)	79 (13)	80 (12)	78 (13)
Systolic blood pressure, mmHg	139 (22)	139 (20)	139 (21)	141 (21)	138 (24)
Albumin, g/dL	4.0 (0.4)	4.1 (0.3)	4.1 (0.3)	4.0 (0.4)	3.9 (0.4)
Haemoglobin, g/dL	12.2 (2.1)	13.2 (1.9)	12.5 (1.8)	11.6 (2.0)	11.6 (2.2)
Phosphate, mmol/L	1.4 (0.5)	1.1 (0.2)	1.2 (0.3)	1.5 (0.4)	1.7 (0.6)
Urinary albumin:creatinine ratio, mg/g	675 (1196)	468 (1012)	624 (1094)	1047 (1486)	-
Total cholesterol, mmol/L	4.9 (1.2)	5.1 (1.1)	5.1 (1.2)	4.8 (1.2)	4.6 (1.2)
HDL-cholesterol, mmol/L	1.1 (0.3)	1.1 (0.3)	1.1 (0.3)	1.1 (0.3)	1.1 (0.3)
Time since CKD diagnosis, years	11.6 (13.4)	9.7 (12.3)	11.6 (14.5)	11.5 (13.4)	12.9 (13.0)
Cause of kidney disease					
Diabetic nephropathy	1363 (15%)	289 (14%)	395 (14%)	201 (14%)	477 (16%)
Cystic kidney disease	1046 (11%)	171 (8%)	286 (10%)	216 (15%)	371 (12%)
Other/unknown	6861 (74%)	1560 (77%)	2086 (75%)	1031 (71%)	2177 (72%)

[1]a small number of participants with eGFR ≥45 ml/min/1.73m^2); 10 participants on kidney transplant at baseline are excluded. RRT, renal replacement therapy; [2] eGFR – estimated Glomerular Filtration Rate (ml/min/1.73m^2).
Results are shown as mean (SD) or N (%), as appropriate.

Table S2 Cardiovascular disease events in SHARP contributing to cardiovascular risk estimation

CKD status and age at baseline	N	Event (per 1000 years of follow-up)[1]		
		Vascular death	MAE or vascular death	MVE or vascular death
Overall	9270	736 (19.1)	1753 (46.0)	1785 (46.9)
<65 years	5395	298 (12.5)	745 (31.5)	769 (32.5)
≥65 years	3875	438 (29.8)	1008 (69.9)	1016 (70.5)
Stage 3B[2]	2020	94 (10.6)	232 (26.3)	238 (27.0)
<65 years	1201	40 (7.3)	96 (17.6)	101 (18.5)
≥65 years	819	54 (15.9)	136 (40.4)	137 (40.7)
Stage 4	2767	175 (14.7)	454 (38.5)	463 (39.3)
<65 years	1374	45 (7.2)	137 (22.1)	142 (22.9)
≥65 years	1393	130 (23.0)	317 (56.9)	321 (57.6)
Stage 5, not on RRT	1448	139 (24.6)	296 (53.1)	300 (53.8)
<65 years	806	47 (13.8)	111 (32.9)	114 (33.8)
≥65 years	642	92 (40.8)	185 (83.8)	186 (84.3)
On dialysis	3025	328 (27.1)	771 (65.0)	784 (66.2)
<65 years	2006	166 (19.1)	401 (46.7)	412 (48.0)
≥65 years	1019	162 (47.9)	370 (113.1)	372 (113.8)

CKD, chronic kidney disease; RRT, renal replacement therapy; MAE, major atherosclerotic event; MVE, major vascular event

[1]First occurrence of the event in each annual period of follow-up contributes to risk estimation; [2]a small number of participants with eGFR ≥45 ml/min/1.73m^2 at entry into the study.

Table S3 Transitions between CKD stages during the 41324 annual periods of follow-up in SHARP

CKD status and age at start of annual period	CKD status or died at end of annual period, N (%)					
	Stage 3B[1]	Stage 4	Stage 5, not on RRT	Kidney transplant	On dialysis	Died
Stage 3B[1]	6676 (80.6%)	1371 (16.6%)	19 (0.2%)	1 (0.0%)	23 (0.3%)	190 (2.3%)
<65 years	4122 (82.6%)	777 (15.6%)	13 (0.3%)	1 (0.0%)	14 (0.3%)	64 (1.3%)
≥65 years	2554 (77.7%)	594 (18.1%)	6 (0.2%)	0 (0.0%)	9 (0.3%)	126 (3.8%)
Stage 4	820 (6.7%)	9275 (75.6%)	1330 (10.8%)	36 (0.3%)	341 (2.8%)	466 (3.8%)
<65 years	374 (6.0%)	4647 (75.0%)	798 (12.9%)	34 (0.5%)	235 (3.8%)	111 (1.8%)
≥65 years	446 (7.3%)	4628 (76.3%)	532 (8.8%)	2 (0.0%)	106 (1.7%)	355 (5.8%)
Stage 5, not on RRT	10 (0.2%)	247 (5.0%)	2788 (56.9%)	159 (3.2%)	1372 (28.0%)	328 (6.7%)
<65 years	7 (0.3%)	104 (3.9%)	1464 (54.2%)	149 (5.5%)	871 (32.3%)	105 (3.9%)
≥65 years	3 (0.1%)	143 (6.5%)	1324 (60.1%)	10 (0.5%)	501 (22.7%)	223 (10.1%)
Kidney transplant	-	-	-	2238 (97.2%)	29[2] (1.3%)	35 (1.5%)
<65 years	-	-	-	2028 (97.5%)	26 (1.3%)	25 (1.2%)
≥65 years	-	-	-	210 (94.2%)	3 (1.3%)	10 (4.5%)
On dialysis	-	-	-	939 (6.0%)	13544 (86.5%)	1176 (7.5%)
<65 years	-	-	-	845 (8%)	9170 (87%)	521 (4.9%)
≥65 years	-	-	-	94 (1.8%)	4374 (85.4%)	655 (12.8%)

CKD, chronic kidney disease; RRT, renal replacement therapy

[1]a small number of participants with eGFR ≥45 ml/min/1.73m^2 at entry into the study; [2]this number excludes kidney transplants that failed within the annual period of transplantation

Table S4 Harrell's C-index assessing discrimination of the SHARP CKD-CVD model

	Vascular death	MVE or vascular death	RRT
	**All SHARP participants, at 5 years**		
	0.77 (0.75-0.79)	0.73 (0.72-0.74)	0.85 (0.85-0.86)
	SHARP participants by CKD stage at baseline		
Stage 3B	0.83 (0.79-0.86)	0.74 (0.71-0.77)	0.80 (0.76-0.83)
Stage 4	0.77 (0.73-0.80)	0.72 (0.70-0.75)	0.76 (0.75-0.78)
Stage 5, not on RRT	0.76 (0.72-0.80)	0.72 (0.69-0.75)	0.65 (0.63-0.67)
On dialysis	0.72 (0.69-0.74)	0.70 (0.68-0.72)	-
	**CRIB participants, at 5 years**		
	0.75 (0.71-0.80)	-	0.84 (0.82-0.86)
	**4D participants, at 4 years**		
	0.61 (0.58-0.64)	0.58 (0.55-0.61)	-
	**AURORA participants, at 3 years**		
	0.65 (0.63-0.67)	0.65 (0.63-0.66)	-

Note: The c-index (ie, Harrell's c-statistic) is a non-parametric rank-correlation measure used for a censored response variable. Predictions for external studies were generated using baseline information on participants in those studies and the SHARP CKD-CVD model; further details are available in Methods and Item S1. A value above 0.5 indicates positive predictive discrimination. The c-index was calculated at the median follow-up in the study. MVE, major vascular event; RRT, renal replacement therapy; CKD, chronic kidney disease.

Table S5 Baseline characteristics of participants in CRIB, 4D and AURORA

	CRIB				4D	AURORA
	Overall	Stage 3B	Stage 4	Stage 5, not on RRT		
	n = 382	n = 79	n = 173	n = 130	n = 1,255	n = 2,773
Age, years	61 (14)	58 (14)	63 (14)	61 (14)	66 (8)	64 (9)
Male	248 (65%)	65 (82%)	107 (62%)	76 (58%)	677 (54%)	1723 (62%)
Ethnicity						
White	336 (88%)	67 (85%)	158 (91%)	111 (85%)	1,255 (100%)[1]	2,354 (85%)
Asian	24 (6%)	4 (5%)	10 (6%)	10 (8%)	0 (0%)	139 (5%)
Black	8 (10%)	5 (3%)	9 (7%)	9 (8%)	0 (0%)	98 (4%)
Other	0 (0%)	0 (0%)	0 (0%)	0 (0%)	0 (0%)	182 (7%)
Smoking status						
Current smoker	48 (13%)	11 (14%)	21 (12%)	16 (12%)	108 (9%)	429 (15%)
Ex-smoker	194 (51%)	43 (54%)	86 (50%)	65 (50%)	n/a	n/a
Previous Vascular Disease	171 (45%)	37 (47%)	76 (44%)	58 (45%)	911 (73%)	1,110 (40%)
Diabetes Mellitus	66 (17%)	13 (16%)	24 (14%)	29 (22%)	1,255 (100%)	731 (26%)
Body-Mass Index, kg/m^2	27 (5)	27 (5)	27 (5)	26 (5)	28 (5)	25 (5)
Diastolic blood pressure, mmHg	84 (12)	87 (11)	84 (12)	82 (11)	76 (11)	76 (13)
Systolic blood pressure, mmHg	152 (22)	150 (23)	152 (24)	152 (21)	146 (22)	137 (24)
Albumin, g/dL	4.2 (0.4)	4.3 (0.4)	4.2 (0.5)	4.1 (0.4)	3.8 (0.3)	4.0 (0.3)
Haemoglobin, g/dL	12.1 (2)	13.7 (1.8)	12.2 (1.8)	10.9 (1.6)	10.9 (1.3)	11.7 (1.6)
Urinary ACR, mg/g	926 (1250)	727 (1182)	719 (1048)	1351 (1446)	-	-
Total cholesterol, mmol/L	5.6 (1.3)	5.8 (1.1)	5.7 (1.4)	5.4 (1.2)	5.7 (1.1)	4.5 (1.1)
HDL-cholesterol, mmol/L	1.3 (0.4)	1.3 (0.4)	1.3 (0.4)	1.2 (0.4)	0.9 (0.3)	1.2 (0.4)

Cause of kidney disease						
Diabetic nephropathy	31 (8%)	4 (5%)	12 (7%)	15 (12%)		
Cystic kidney disease	33 (9%)	5 (6%)	12 (7%)	16 (12%)	n/a	n/a
Other/unknown	318 (83%)	70 (89%)	149 (86%)	99 (76%)		
Duration of RRT, years	-	-	-	-	0.7 (0.6)	4.4 (5.2)

[1]No data on ethnicity was available in 4D, white ethnicity was assumed in simulations. RRT, renal replacement therapy; ACR, albumin:creatinine ratio; n/a not available.

Results are shown as mean (SD) or N (%), as appropriate.

Table S6 Predicted life expectancy and quality adjusted life years for participants in SHARP, by CKD stage at randomisation

CKD stage at baseline	Age at baseline, years									
	Men					Women				
	<50	50-59	60-69	70-79	≥80	<50	50-59	60-69	70-79	≥80
	**Predicted life expectancy, years (95% CI)**									
Stage 3B[1]	24.1 (23.5, 24.7)	18.3 (17.9, 18.7)	13.5 (13.2, 13.9)	9.5 (9.2, 9.8)	6.4 (6.2, 6.6)	24.0 (23.3, 24.6)	19.8 (19.3, 20.3)	14.8 (14.4, 15.3)	10.7 (10.4, 11.0)	7.3 (7.1, 7.4)
Stage 4	22.0 (21.3, 22.7)	15.4 (15.0, 15.9)	11.6 (11.3, 11.9)	8.2 (7.9, 8.4)	5.4 (5.2, 5.5)	22.3 (21.6, 23.0)	17.2 (16.7, 17.7)	12.5 (12.1, 12.8)	9.4 (9.1, 9.6)	6.1 (5.9, 6.2)
Stage 5, not on RRT	19.9 (18.9, 20.9)	12.7 (12.1, 13.3)	8.3 (8.0, 8.7)	5.9 (5.6, 6.1)	3.8 (3.7, 4.0)	20.0 (19.1, 20.9)	12.7 (12.2, 13.3)	9.1 (8.7, 9.5)	6.3 (6.0, 6.6)	4.7 (4.5, 4.9)
On dialysis	18.5 (17.8, 19.3)	12.1 (11.7, 12.6)	7.5 (7.2, 7.8)	4.8 (4.6, 5.0)	3.2 (3.1, 3.3)	18.2 (17.5, 19.0)	12.0 (11.6, 12.5)	7.8 (7.5, 8.1)	5.3 (5.1, 5.5)	3.5 (3.3, 3.6)
	**Predicted quality-adjusted life years [QALYs] (95% CI)**									
Stage 3B[1]	20.9 (20.4, 21.4)	15.1 (14.8, 15.5)	10.6 (10.3, 10.9)	7.1 (6.9, 7.4)	4.7 (4.5, 4.8)	19.4 (18.8, 20.0)	15.2 (14.7, 15.6)	10.7 (10.3, 11.1)	7.3 (7.1, 7.6)	4.8 (4.7, 5.0)
Stage 4	19.0 (18.4, 19.6)	12.6 (12.3, 13.0)	9.1 (8.8, 9.3)	6.1 (5.9, 6.3)	3.8 (3.7, 4.0)	18.0 (17.4, 18.6)	13.3 (12.8, 13.7)	9.0 (8.7, 9.3)	6.4 (6.2, 6.6)	4.0 (3.9, 4.2)
Stage 5, not on RRT	17.3 (16.4, 18.1)	10.4 (10.0, 10.9)	6.4 (6.2, 6.7)	4.3 (4.1, 4.5)	2.7 (2.6, 2.8)	16.2 (15.5, 16.9)	9.7 (9.3, 10.2)	6.5 (6.3, 6.9)	4.3 (4.1, 4.5)	3.1 (2.9, 3.2)
On dialysis	15.6 (15.0, 16.3)	9.8 (9.4, 10.1)	5.6 (5.4, 5.9)	3.4 (3.2, 3.5)	2.2 (2.1, 2.3)	14.3 (13.8, 14.9)	9.0 (8.6, 9.3)	5.4 (5.2, 5.6)	3.4 (3.3, 3.6)	2.2 (2.0, 2.3)

	**Predicted reduction in life expectancy [years] following a nonfatal major atherosclerotic event[2] (95% CI)**									
Stage 3B[1]	2.5 (1.0, 4.1)	2.2 (1.1, 3.3)	1.7 (0.8, 2.5)	1.1 (0.5, 1.7)	0.6 (0.3, 0.9)	2.9 (1.4, 4.4)	2.6 (1.4, 3.8)	2.0 (1.0, 2.9)	1.2 (0.6, 1.8)	0.5 (0.2, 0.8)
Stage 4	2.2 (0.8, 3.6)	2.2 (1.3, 3.2)	2.0 (1.3, 2.6)	1.3 (0.8, 1.7)	0.8 (0.5, 1.0)	2.9 (1.5, 4.4)	2.8 (1.7, 3.9)	2.2 (1.4, 2.9)	1.5 (1.0, 2.0)	0.7 (0.4, 1.0)
Stage 5, not on RRT	1.8 (0.2, 3.4)	1.5 (0.6, 2.4)	1.4 (0.8, 1.9)	1.1 (0.7, 1.4)	0.7 (0.5, 0.8)	2.0 (0.4, 3.6)	1.8 (0.8, 2.7)	1.7 (1.0, 2.3)	1.4 (0.9, 1.8)	0.9 (0.6, 1.1)
On dialysis	1.4 (0.0, 2.9)	1.2 (0.2, 2.1)	0.9 (0.3, 1.4)	0.7 (0.4, 1.0)	0.5 (0.3, 0.7)	1.3 (-0.1, 2.8)	1.0 (0.0, 1.9)	0.8 (0.3, 1.4)	0.7 (0.3, 1.0)	0.5 (0.3, 0.7)
	**Predicted reduction in quality-adjusted life years [QALYs] following a nonfatal major atherosclerotic event[2] (95% CI)**									
Stage 3B[1]	4.1 (2.8, 5.3)	3.3 (2.2, 4.2)	2.3 (1.6, 3.0)	1.5 (1.1, 1.9)	0.9 (0.7, 1.2)	4.3 (3.1, 5.4)	3.5 (2.5, 4.5)	2.6 (1.8, 3.2)	1.6 (1.1, 2.0)	0.9 (0.6, 1.1)
Stage 4	3.7 (2.5, 4.8)	3.0 (2.2, 3.7)	2.4 (1.8, 2.9)	1.5 (1.2, 1.9)	0.9 (0.7, 1.1)	4.2 (3.0, 5.3)	3.5 (2.6, 4.2)	2.5 (2.0, 3.1)	1.7 (1.3, 2.1)	0.9 (0.7, 1.1)
Stage 5, not on RRT	3.2 (1.9, 4.5)	2.2 (1.4, 2.9)	1.7 (1.2, 2.1)	1.2 (1.0, 1.5)	0.7 (0.6, 0.9)	3.3 (2.1, 4.5)	2.4 (1.6, 3.1)	1.9 (1.4, 2.3)	1.4 (1.1, 1.7)	0.9 (0.8, 1.1)
On dialysis	2.7 (1.5, 3.8)	1.8 (1.1, 2.6)	1.2 (0.8, 1.6)	0.8 (0.6, 1.0)	0.6 (0.5, 0.7)	2.5 (1.5, 3.6)	1.7 (1.0, 2.3)	1.1 (0.7, 1.5)	0.8 (0.6, 1.0)	0.6 (0.4, 0.7)

CKD, chronic kidney disease; RRT, renal replacement therapy; CI, confidence interval
The predictions are for SHARP participants in the absence of lipid-lowering treatment
[1]a small number of participants with eGFR ≥45 ml/min/1.73m^2
[2]The major atherosclerotic event is simulated to occur in year prior to entry in the model.

Item S1 Supplementary methods

Specification of covariates in CVD and CKD submodels

Continuous covariates were categorised into three groups defined by the approximate tertiles of the distributions, with missing data assigned to a separate group (or, if missing for <5% of participants, into the middle group). Age, sex, ethnicity, smoking, uACR and cardiovascular and kidney disease history were retained in all risk equations regardless of their statistical significance. P-values <0.01 were considered significant. Missing data (Table SM1) were either assigned into a separate category (for albumin, haemoglobin, phosphate and UACR), or, if data were missing for fewer than 5% of participants, to the middle group (≥25, <30 kg/m^2 for BMI; ≥75, <85 mmHg for diastolic blood pressure; ≥130, <150 mmHg for systolic blood pressure; and ≥0.9, 1.2 mmol/L for HDL cholesterol).

Table SM1 Missing biomarkers data at entry into SHARP

Characteristic	Number of participants with missing data (%)
BMI	200 (2%)
Diastolic blood pressure	17 (0%)
Systolic blood pressure	16 (0%)
Albumin	763 (8%)
Haemoglobin	1,405 (15%)
Phosphate	1,652 (18%)
Urinary ACR	669 (7%)
HDL cholesterol	381 (4%)

BMI, body-mass index; ACR, albumin:creatinine ratio

For participants not on RRT, their eGFR was calculated from creatinine using the CKD-EPI equation[1], and the mean eGFR during that year determined the CKD stage. For a small (<2%) number of participants, no follow-up visits were recorded in some annual periods during which they were known to be alive, and therefore information on their CKD status could not be extracted. For each participant it was known whether they were on RRT at the time; whether they were on transplant, dialysis or pre-RRT at randomisation; and – if they reached

RRT during the study - their first RRT modality. If a participant was not on RRT during a period with no data, their latest CKD status was carried until the next available information. If no further information was available until the end of the trial, the latest CKD status was carried for two years, after which the participant was assumed to be lost to attrition. For participants on RRT, the latest information on modality was carried over until the next available information or end of the follow-up.

Risk equations in the CVD submodel

The annual risks of three nested composite cardiovascular endpoints were evaluated: 1) vascular death (defined as coronary, stroke or other vascular death), 2) vascular death or nonfatal major atherosclerotic event (ie, vascular death, myocardial infarction, non-haemorrhagic stroke, or arterial revascularisation), and 3) vascular death or nonfatal major vascular event (ie, vascular death, myocardial infarction, any stroke or arterial revascularisation). For each participant, the annual risks of these cardiovascular endpoints were estimated using survival risk equations with a range of baseline characteristics and annually updated age, time since CKD diagnosis, cardiovascular disease history and CKD status. The Andersen-Gill generalisation of the Cox proportional hazards model was used with all potential covariates included[2,3], as both external evidence[4] and the Cox modelling confirmed the suitability of the proportional hazards methods An automatic forward- and backwards-selection procedure based on minimum Akaike Information Criterion (AIC) was adopted[5]. Finally, the variables judged not significant, both statistically and clinically, were removed one-by-one, and the resulting models were compared to the model without the excluded variable using the likelihood ratio test. Parametric proportional hazard survival models, including selected variables, were then estimated to support extrapolation over a patient’s lifetime. Exponential, Weibull and Gompertz proportional hazards models were

considered, as both external evidence[4] and the Cox modelling confirmed the suitability of the proportional hazards methods. The appropriate survival distribution was selected using AIC[5].

The risk equations' parameters, estimated following multiple imputation of missing data, were similar.

Risk equations in the CKD submodel

The procedure for covariate selection in the CKD risk equations (a multinomial logistic regression in pre-RRT and a logistic regression for transitions from dialysis to transplantation) was analogous to that used in the Cox proportional hazards models behind the CVD submodel. The variables judged not significant were removed one-by-one, and the two models were compared using the likelihood ratio test.

The risk equations' parameters, estimated following multiple imputation of missing data, were similar.

To account for multiple endpoints per participant, robust standard errors were estimated[6]. In sensitivity analyses, the risk equations' parameters were compared to corresponding parameter estimates following a multiple imputation approach for missing data[7].

SHARP CKD-CVD model

The CVD and CKD submodels were combined into the *SHARP CKD-CVD model,* a first-order Markov model with an annual cycle of transition (Figure 1). The input to the model consists of individual participant's characteristics collected at baseline. Additionally, at the start of each cycle, participant's age and CKD duration are updated, and information on the contemporaneous CKD stage and most recent CVD event is extracted. The information initially feeds into the CKD submodel, which updates the participant's CKD stage for the annual period. This, in turn, provides the input for the CVD submodel, and the participant's

CVD status in the year is updated. The model then enters into the next annual cycle, where the updated disease history is used.

The model of CVD in CKD distinguishes between atherosclerotic cardiovascular disease (myocardial infarction or coronary death, non-haemorrhagic stroke or arterial revascularisation excluding dialysis access procedures) and vascular non-atherosclerotic disease (haemorrhagic stroke or cardiac non-coronary death), which could be differently influenced by interventions. Therefore, the CVD submodel includes three non-fatal states: no vascular events, non-fatal major atherosclerotic event, and non-fatal haemorrhagic stroke; and two (absorbing) fatal states: non-vascular death and vascular death. To reflect immediate increase and subsequent decrease in risk of adverse outcomes associated with recent cardiovascular events, additional tunnel states are used to track duration since latest occurrence of non-fatal major atherosclerotic event (<1 year, 1-2 and ≥2 years since the last event). In this submodel, during each annual period events were simulated in the following order: non-vascular death, vascular death, nonfatal major atherosclerotic event or nonfatal haemorrhagic stroke. For each participant, the first occurrence of each endpoint during each year of follow-up contributed to risk estimation. Specifically, once an event of the required kind occurred, information from the remaining part of the year was not used, with information from start of the following year contributing to estimations. Annual non-vascular mortality rates derived from UK population data (Table SM2) were implemented in the model.

The choice of the pre-RRT states in the model of CKD progression was based on the recommended Kidney Disease Improving Global Outcomes (KDIGO) GFR categories[8]. The recommended KDIGO ACR categories (<30 mg/g, 30-300 mg/g, >300mg/g) were not used in classification as information on annual UACR was not collected. Instead, the baseline value of UACR was used as a covariate in all risk equations. Possible directions of transitions

between states were based on transitions observed in SHARP. In the RRT states, kidney transplant was modelled separately from dialysis due to known associations with better outcomes[9]; probability of transplant failing within the year of transplantation was based on SHARP data. The CKD submodel includes three pre-RRT states (CKD stage 3B, CKD stage 4 and CKD stage 5 not on RRT) and two RRT states (on dialysis, with a functioning kidney transplant). Additional tunnel states are employed to account for the increased risk of adverse outcomes associated with longer RRT (<1 year, 1-2, 2-3 and ≥3 years since initiation of renal replacement therapy).

Together, the model states comprise all possible combinations of non-fatal CVD and CKD states as well as the two fatal (absorbing) states: vascular and non-vascular death.

Transitional probabilities are calculated as follows:

a) For all survival data risk equations, used in the CVD submodel, the annual transitional probability in year *t* is calculated as:

$$p(t) = 1 - \exp[H(365*(t-1)) - H(365*t)],$$

where H(t) denotes the cumulative hazard at the end of year *t*. This, in turn, is calculated depending on the survival model. For the exponential model, the cumulative hazard at time t is H(t)= λt, and for the Gompertz model, H(t) = $\lambda \gamma^{-1}$ (exp(γt) – 1). Here, λ is $\exp(\sum_j \beta_j x_j)$, β_j a vector of regression coefficients, x_j is a vector of covariates and γ is the ancillary parameter in the Gompertz model. A hierarchy of events is imposed on the model so that the probabilities of cardiovascular events (or non-vascular death) are extracted as follows:

(1) Nonvascular death (NVD): p(NVD) as per Table S1

(2) Vascular death (VD), conditional on not experiencing (1):

p = p(VD)*[1 – p(NVD)]

(3) Nonfatal major atherosclerotic event (MAE), conditional on not experiencing (1)-(2):

p = max[0, p(MAE or VD) – p(VD)] * [1– p(NVD)]

(4) Nonfatal haemorrhagic stroke, conditional on non-experiencing (1)-(3):

p = max[0, p(MVE or VD) – max[p(MAE or VD), p(VD)]] * [1– p(NVD)]

b) For the multinomial log-linear regression (used for transition from a pre-RRT state to any CKD state), the probability of remaining in the category *i* is:

$$\mathrm{tp}(Y = i, t) = \frac{1}{1 + \sum_{k \neq i} \exp(\sum_j \beta_{kj} x_j)},$$

and the probability of moving into another category i' is:

$$\mathrm{tp}(Y = i', t) = \frac{\exp(\sum_j \beta_{i'j} x_j)}{1 + \sum_{k \neq i} \exp(\sum_j \beta_{kj} x_j)}.$$

c) For the logistic regression (used for transition from ‘on dialysis’ state into ‘on kidney transplant’)

$$\mathrm{tp}(t) = \frac{\exp(\sum_j \beta_j x_j)}{1 + \exp(\sum_j \beta_j x_j)}.$$

Estimation of parameter uncertainty

The parameter uncertainty in all model equations was assessed using bootstrap methods whereby the SHARP individual participant data were bootstrapped and all equations re-estimated on the bootstrap sample data to derive sets of correlated regression coefficients. Parameter uncertainty was propagated in the model by using these sets of estimated coefficients to run the model and evaluate outcomes. This procedure was repeated 1,000 times, and the confidence intervals for the model outcomes evaluated using the equal-tailed percentile method.

External model validation

For the purpose of validation, non-vascular death in the model was simulated using study-specific Cox proportional hazards equations The predictions were simulated in the absence of study lipid-lowering treatments, and the following assumptions were made for baseline covariates not available in the external study datasets based on most likely value in SHARP: CRIB participants were simulated to have adult dependants, be educated to a GCSE/vocational level and have CKD diagnosis for 10.9 years; 4D participants were simulated of white ethnicity, be educated to a GCSE/vocational level, without previous transplant, with diabetic nephropathy, and with current non-smokers identical to never smokers in SHARP; AURORA participants were simulated to have adult dependants, be educated to a GCSE/vocational level, without previous transplant, with other (ie not diabetic nephropathy or cystic kidney disease) cause of CKD, and with current non-smokers identical to never smokers in SHARP).

Due to large differences in rates of transplantation in SHARP and the external studies, transplantation rates in the model for the purpose of validation were calibrated to correspond to those in the respective studies. The validation algorithm therefore was performed in two stages: firstly, the results of the initial simulation of outcomes for participants from an external dataset were obtained. Subsequently, a transplantation calibration factor is calculated as a ratio of observed versus predicted rates of kidney transplantations in the study. The simulation is then re-run, with the transplantation calibration factor applied to the probability of receiving a transplant, so that the probability of receiving a transplant multiplied by the transplantation calibration factor. The transplantation calibration factors for the CRIB, 4D and AURORA study were, respectively, 1.7, 0.2 and 0.9. No further calibration was performed for the purpose of validating model predictions against rates of events in external studies.

The following adjustments were performed to allow comparison of SHARP CKD-CVD model performance with other risk scores. Since the Pooled Cohort Equations predict the risk of the first atherosclerotic cardiovascular disease event (ASCVD), while the first major vascular event or vascular death endpoint in SHARP, 4D and AURORA also include revascularisations and non-ASCVD vascular deaths, the risks produced by the Pooled Cohort Equations were calibrated by a factor corresponding to the proportion of revascularisations and non-ASCVD vascular deaths in the respective group of participants. The calibrating factors were 0.7 for SHARP participants with CKD stage 3B, 4 and 5 and 0.6 for SHARP participants on dialysis at randomisation; 0.7 for 4D participants and 0.7 for AURORA participants.

Table SM2 Annual probabilities of non-vascular death in SHARP CKD-CVD model

Age	Men					Women				
	Stage 3	Stage 4	Stage 5, not on RRT	Transplant	Dialysis	Stage3	Stage 4	Stage 5, not on RRT	Transplant	Dialysis
40-44	0.3%	0.4%	0.5%	0.8%	2.8%	0.2%	0.2%	0.3%	0.9%	3.3%
45-49	0.4%	0.6%	0.7%	0.8%	2.8%	0.3%	0.4%	0.5%	0.9%	3.3%
50-54	0.4%	0.6%	0.7%	1.3%	4.6%	0.3%	0.4%	0.5%	0.8%	5.9%
55-59	1.0%	1.3%	1.7%	1.6%	5.2%	0.6%	0.8%	1.1%	1.0%	6.6%
60-64	1.0%	1.3%	1.7%	2.8%	7.2%	0.6%	0.8%	1.1%	2.4%	8.1%
65-69	1.8%	2.6%	3.7%	3.6%	9.5%	1.1%	1.6%	2.3%	4.0%	8.9%
70-74	1.8%	2.6%	3.7%	5.7%	10.8%	1.1%	1.6%	2.3%	5.1%	10.7%
75-79	4.1%	5.7%	9.3%	7.0%	14.7%	2.9%	4.1%	6.7%	6.6%	12.7%
80-84	4.1%	5.7%	9.3%	12.6%	16.3%	2.9%	4.1%	6.7%	11.6%	17.8%
85+	9.5%	12.4%	22.6%	12.6%	24.5%	8.5%	11.1%	20.4%	11.6%	22.1%

CKD, chronic kidney disease; RRT, renal replacement therapy

Non-vascular mortality rates were derived from UK-wide data. For those on dialysis or on kidney transplant, 2011 mortality data were obtained from the UK renal registry by age and gender[1]. For pre-RRT stages, age- and gender- specific all-cause mortality rates of general population[2-4] were adjusted using the increased hazard associated with CKD[5,6]. The non-vascular mortality rates were calculated assuming ratio of vascular to non-vascular mortality as reported in the MRC Older People study[7].

1. Feest TG FD, MacPhee I, Sinha MD, Udayaraj U, Wilkie M, Williams AJ (2012) UK Renal Registry 2012, 15th Annual Report of the Renal Association. Bristol, UK.
2. Office for National Statistics (2010). Mortality Statistics: Deaths Registered in England and Wales (Series DR) http://www.ons.gov.uk/ons/rel/vsob1/mortality-statistics--deaths-registered-in-england-and-wales--series-dr-/2010/index.html. Accessed 03 Apr 2013.
3.General Register Office for Scotland (2010) Vital Events Reference Tables. http://www.gro-scotland.gov.uk/statistics/theme/vital-events/general/ref-tables/archive/2010/deaths.html. Accessed 03 Apr 2013.
4. Northern Ireland Statistics & Research Agency (2010) Vital Statistics (Deaths). http://www.nisra.gov.uk/demography/default.asp10.htm. Accessed 03 Apr 2013.
5. de Lusignan S, Chan T, Gallagher H, van Vlymaen J, Thomas N, et al. (2009) Chronic kidney disease management in southeast England: a preliminary cross-sectional report from the QICKD–quality improvement in chronic kidney disease study. Prim Care Cardiovasc J 33(9):33-9.
6. O'Hare AM, Bertenthal D, Covinsky KE, Landefeld CS, Sen S, et al. (2006) Mortality risk stratification in chronic kidney disease: one size for all ages? JASN 17(3):846-853.
7. Chronic Kidney Disease Prognosis Consortium (2010). Association of estimated glomerular filtration rate and albuminuria with all-cause and cardiovascular mortality in general population cohorts: a collaborative meta-analysis. Lancet 375(9731):2073-2081.

All analyses were performed using R 3.0.2[10] or Stata 12.1[11]. The Figures were produced using the ggplot2 plotting system[12]. The web interface for the model was created using the Shiny web application framework[13].

Supplementary references

1. Levey AS, Stevens LA, Schmid CH, et al. A new equation to estimate glomerular filtration rate. *Ann Intern Med.* 2009;150(9):604-612.
2. Cox DR. Regression models and life-tables. *J Roy Statist Soc Ser B.* 1972;34:187--220.
3. Andersen PK, Gill RD. Cox's regression model for counting processes: a large sample study. *Ann Statist.* 1982;10(4):1100-1120.
4. Hippisley-Cox J, Coupland C, Vinogradova Y, Robson J, May M, Brindle P. Derivation and validation of QRISK, a new cardiovascular disease risk score for the United Kingdom: prospective open cohort study. *BMJ.* 2007;335(7611):136.
5. Akaike H. A new look at the statistical model identification. *IEEE Transactions on Automatic Control.* 1974;19:716-723.
6. Gould WW, Pitblado JS, Sribney WM. *Maximum Likelihood Estimation with Stata.* 3 ed. College Station, TX: Stata Press; 2006.
7. Schafer JL. *Analysis of incomplete multivariate data.* Chapman & Hall/CRC; 1997.
8. Kidney Disease: Improving Global Outcomes (KDIGO) CKD Work Group. KDIGO 2012 Clinical Practice Guideline for the Evaluation and Management of Chronic Kidney Disease. *Kidney Int.* 2013;Suppl.(3):150.
9. Tonelli M, Wiebe N, Knoll G, et al. Systematic review: kidney transplantation compared with dialysis in clinically relevant outcomes. *Am J Transplant.* 2011;11(10):2093-2109.
10. *R: A Language and Environment for Statistical Computing* [computer program]. R Foundation for Statistical Computing, Vienna, Austria; 2008.
11. StataCorp. 2011. Stata Statistical Software: Release 12.
12. Wickham H. *ggplot2: elegant graphics for data analysis.* Springer New York; 2009.
13. *Shiny: Web Application Framework for R* [computer program]. 2016.
